# Butyl­triethyl­ammonium tetra­chlorido­ferrate(III)

**DOI:** 10.1107/S1600536812017047

**Published:** 2012-04-21

**Authors:** Lei Jin, Yong-Jun Li

**Affiliations:** a College of Chemistry and Chemical Engineering, Southeast University, Nanjing 210096, People’s Republic of China

## Abstract

In the title compound, (C_10_H_24_N)[FeCl_4_], no classical hydrogen bonds are observed. The butyl­triethyl­ammonium cations inter­act with the tetra­hedral [FeCl_4_]^−^ anion through weak C—H⋯Cl inter­actions, forming a three-dimensional network.

## Related literature

For background to mol­ecular–ionic and ferroelectric–dielectric compounds, see: Hay & Geib (2005 ▶); Zhang *et al.* (2010 ▶).
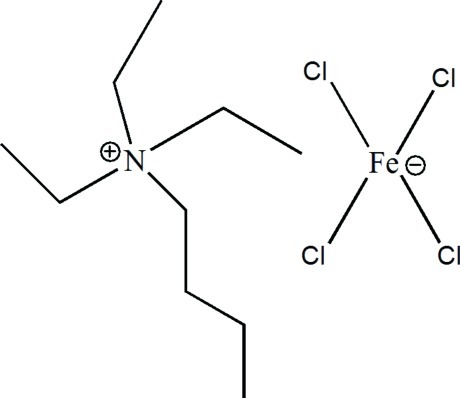


## Experimental

### 

#### Crystal data

(C_10_H_24_N)[FeCl_4_]
*M* *_r_* = 355.95
Monoclinic, 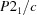

*a* = 7.6197 (15) Å
*b* = 11.671 (2) Å
*c* = 18.473 (4) Å
β = 91.51 (3)°
*V* = 1642.2 (6) Å^3^
*Z* = 4
Mo *K*α radiation
μ = 1.55 mm^−1^
*T* = 293 K
0.28 × 0.24 × 0.20 mm

#### Data collection

Rigaku Mercury2 diffractometer
Absorption correction: multi-scan (*CrystalClear*; Rigaku, 2005 ▶) *T*_min_ = 0.655, *T*_max_ = 0.734
16819 measured reflections
3754 independent reflections
2766 reflections with *I* > 2σ(*I*)
*R*_int_ = 0.052

#### Refinement

*R*[*F* ^2^ > 2σ(*F* ^2^)] = 0.048
*wR*(*F* ^2^) = 0.117
*S* = 1.10
3754 reflections
149 parameters
H-atom parameters constrained
Δρ_max_ = 0.30 e Å^−3^
Δρ_min_ = −0.53 e Å^−3^

### 

Data collection: *CrystalClear* (Rigaku, 2005 ▶); cell refinement: *CrystalClear*; data reduction: *CrystalClear*; program(s) used to solve structure: *SHELXS97* (Sheldrick, 2008 ▶); program(s) used to refine structure: *SHELXL97* (Sheldrick, 2008 ▶); molecular graphics: *SHELXTL* (Sheldrick, 2008 ▶); software used to prepare material for publication: *SHELXTL*.

## Supplementary Material

Crystal structure: contains datablock(s) I, global. DOI: 10.1107/S1600536812017047/ez2288sup1.cif

Structure factors: contains datablock(s) I. DOI: 10.1107/S1600536812017047/ez2288Isup2.hkl

Additional supplementary materials:  crystallographic information; 3D view; checkCIF report

## Figures and Tables

**Table 1:** Hydrogen-bond geometry (Å, °)

*D*—H⋯*A*	*D*—H	H⋯*A*	*D*⋯*A*	*D*—H⋯*A*
C3—H3*B*⋯Cl3^i^	0.96	2.87	3.790 (4)	162
C4—H4*A*⋯Cl1	0.97	2.92	3.859 (3)	163

Symmetry code: (i) 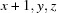.

**Fig. 1.**
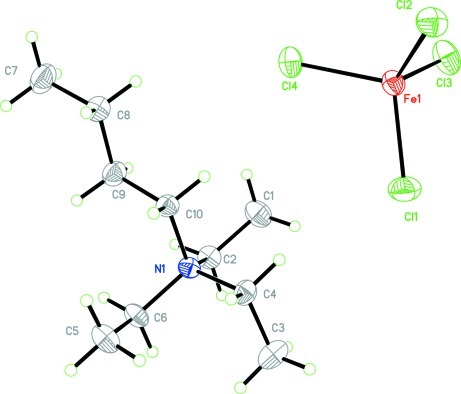
The molecular structure of the title compound, showing the atomic numbering scheme with 30% probability displacement ellipsoids.

## supplementary crystallographic information

### Comment

In our laboratory, we synthesize simple molecular–ionic compounds containing organic-ammonium cations and anions due to the tunability of their special structural features and their ferroelectric-dielectric properties (Hay & Geib, 2005; Zhang *et al.*, 2010). Herein, the crystal structure of the title compound is reported.

The asymmetric unit of the title compound, (C_10_H_24_N^+^).(FeCl_4_^-^) consists of one tetrachloroferrate anion unit and one butyltriethylammonium cation (Fig 1). In the structure, the Fe^III^ ion adopts a distorted tetrahedral geometry surrounded by four Cl^-^ anions with the Fe—Cl bond distances being in the range of 2.1611 (9)–2.1823 (10)? and the Cl–Fe–Cl bond angles being in the range of 109.26 (5)–110.18 (5) (5)°. There are no classic hydrogen bonds found, although weak intermolecular C—H···Cl interactions link the butyltriethylammonium cations and the tetrahedral FeCl_4_^-^ anion into a three-dimensional network.

### Experimental

In room temperaturebutyltriethylammonium(5 mmol,1.17 g) were dissolved in 30 ml water, then FeCl_3_.6H_2_O (5 mmol, 1.35 g) was added into the previous solution slowly with properly sirring. An orange solid appeared immediately and the solid was collected by filtration. Plate orange single crystals suitable for X-ray structure analysis were obtained by the slow evaporation of the above filtrate after a week in air.

The dielectric constant of the compound as a function of temperature indicates that the permittivity is basically temperature-independent (ε = C/(T–T_0_)), suggesting that this compound is not ferroelectric or there may be no distinct phase transition occurring within the measured temperature (below the melting point).

### Refinement

H atoms were placed in calculated positions C—H = 0.96 Å and 0.97 Å for C*sp^3^* atoms), assigned fixed *U*_iso_ values [*U*_iso_ = 1.2*U*eq(C*sp^2^*/N) and 1.5*U*eq(C*sp^3^*)] and allowed to ride.

### Crystal data

**Table:**

(C_10_H_24_N)[FeCl_4_]	*Z* = 4
*M**_r_* = 355.95	*F*(000) = 740
Monoclinic, *P*2_1_/*c*	*D*_x_ = 1.440 Mg m^−^^3^
Hall symbol: -P 2ybc	Mo *K*α radiation, λ = 0.71073 Å
*a* = 7.6197 (15) Å	θ = 3.2–27.5°
*b* = 11.671 (2) Å	µ = 1.55 mm^−^^1^
*c* = 18.473 (4) Å	*T* = 293 K
β = 91.51 (3)°	Block, orange
*V* = 1642.2 (6) Å^3^	0.28 × 0.24 × 0.20 mm

### Data collection

**Table:**

Rigaku Mercury2 diffractometer	3754 independent reflections
Radiation source: fine-focus sealed tube	2766 reflections with *I* > 2σ(*I*)
Graphite monochromator	*R*_int_ = 0.052
Detector resolution: 13.6612 pixels mm^-1^	θ_max_ = 27.5°, θ_min_ = 3.2°
CCD_Profile_fitting scans	*h* = −9→9
Absorption correction: multi-scan (*CrystalClear*; Rigaku, 2005)	*k* = −15→15
*T*_min_ = 0.655, *T*_max_ = 0.734	*l* = −23→23
16819 measured reflections	

### Refinement

**Table:**

Refinement on *F*^2^	Primary atom site location: structure-invariant direct methods
Least-squares matrix: full	Secondary atom site location: difference Fourier map
*R*[*F*^2^ > 2σ(*F*^2^)] = 0.048	Hydrogen site location: inferred from neighbouring sites
*wR*(*F*^2^) = 0.117	H-atom parameters constrained
*S* = 1.10	*w* = 1/[σ^2^(*F*_o_^2^) + (0.0447*P*)^2^ + 0.8101*P*] where *P* = (*F*_o_^2^ + 2*F*_c_^2^)/3
3754 reflections	(Δ/σ)_max_ < 0.001
149 parameters	Δρ_max_ = 0.30 e Å^−^^3^
0 restraints	Δρ_min_ = −0.53 e Å^−^^3^

### Special details

**Table:**

Geometry. All e.s.d.'s (except the e.s.d. in the dihedral angle between two l.s. planes) are estimated using the full covariance matrix. The cell e.s.d.'s are taken into account individually in the estimation of e.s.d.'s in distances, angles and torsion angles; correlations between e.s.d.'s in cell parameters are only used when they are defined by crystal symmetry. An approximate (isotropic) treatment of cell e.s.d.'s is used for estimating e.s.d.'s involving l.s. planes.
Refinement. Refinement of *F*^2^ against ALL reflections. The weighted *R*-factor *wR* and goodness of fit *S* are based on *F*^2^, conventional *R*-factors *R* are based on *F*, with *F* set to zero for negative *F*^2^. The threshold expression of *F*^2^ > σ(*F*^2^) is used only for calculating *R*-factors(gt) *etc*. and is not relevant to the choice of reflections for refinement. *R*-factors based on *F*^2^ are statistically about twice as large as those based on *F*, and *R*- factors based on ALL data will be even larger.

### Fractional atomic coordinates and isotropic or equivalent isotropic displacement parameters (Å^2^)

**Table:**

	*x*	*y*	*z*	*U*_iso_*/*U*_eq_	
C1	0.3896 (4)	0.6311 (3)	0.58003 (19)	0.0585 (9)	
H1A	0.3166	0.6969	0.5712	0.088*	
H1B	0.3229	0.5726	0.6033	0.088*	
H1C	0.4312	0.6025	0.5349	0.088*	
C2	0.5419 (4)	0.6641 (2)	0.62761 (16)	0.0415 (7)	
H2A	0.6114	0.5960	0.6375	0.050*	
H2B	0.4974	0.6906	0.6734	0.050*	
C3	0.8471 (5)	0.6102 (3)	0.5330 (2)	0.0643 (10)	
H3A	0.9425	0.6209	0.5673	0.096*	
H3B	0.8928	0.5938	0.4862	0.096*	
H3C	0.7754	0.5475	0.5481	0.096*	
C4	0.7386 (4)	0.7171 (3)	0.52879 (16)	0.0424 (7)	
H4A	0.6438	0.7052	0.4935	0.051*	
H4B	0.8112	0.7787	0.5110	0.051*	
C5	0.9389 (4)	0.8585 (3)	0.63699 (19)	0.0554 (9)	
H5A	0.9974	0.8332	0.5944	0.083*	
H5B	1.0227	0.8654	0.6765	0.083*	
H5C	0.8849	0.9315	0.6278	0.083*	
C6	0.8021 (4)	0.7737 (3)	0.65603 (16)	0.0441 (7)	
H6A	0.7472	0.7990	0.7001	0.053*	
H6B	0.8589	0.7010	0.6664	0.053*	
C7	0.2880 (5)	1.0847 (3)	0.6797 (2)	0.0670 (10)	
H7A	0.2027	1.0362	0.7017	0.101*	
H7B	0.2314	1.1524	0.6612	0.101*	
H7C	0.3765	1.1057	0.7152	0.101*	
C8	0.3716 (4)	1.0216 (3)	0.61889 (18)	0.0512 (8)	
H8A	0.4515	1.0728	0.5947	0.061*	
H8B	0.2811	0.9988	0.5839	0.061*	
C9	0.4705 (4)	0.9172 (3)	0.64418 (17)	0.0473 (8)	
H9A	0.5563	0.9387	0.6815	0.057*	
H9B	0.3897	0.8629	0.6649	0.057*	
C10	0.5615 (4)	0.8626 (2)	0.58253 (15)	0.0372 (6)	
H10A	0.6427	0.9180	0.5631	0.045*	
H10B	0.4744	0.8461	0.5447	0.045*	
Cl1	0.44676 (12)	0.65139 (8)	0.36000 (6)	0.0695 (3)	
Cl2	0.19519 (11)	0.85280 (7)	0.25806 (4)	0.0545 (2)	
Cl3	−0.01438 (13)	0.61475 (8)	0.33742 (5)	0.0689 (3)	
Cl4	0.15637 (12)	0.84172 (8)	0.44790 (4)	0.0573 (2)	
Fe1	0.19494 (6)	0.73961 (4)	0.35064 (2)	0.04185 (15)	
N1	0.6607 (3)	0.75470 (18)	0.59891 (11)	0.0334 (5)	

### Atomic displacement parameters (Å^2^)

**Table:**

	*U*^11^	*U*^22^	*U*^33^	*U*^12^	*U*^13^	*U*^23^
C1	0.0511 (19)	0.062 (2)	0.062 (2)	−0.0149 (16)	−0.0058 (17)	0.0060 (18)
C2	0.0437 (16)	0.0405 (16)	0.0406 (16)	−0.0044 (13)	0.0042 (13)	0.0069 (13)
C3	0.070 (2)	0.054 (2)	0.070 (2)	0.0133 (18)	0.019 (2)	−0.0038 (18)
C4	0.0472 (17)	0.0459 (17)	0.0345 (16)	0.0019 (13)	0.0081 (13)	−0.0029 (13)
C5	0.0479 (19)	0.063 (2)	0.054 (2)	−0.0124 (16)	−0.0105 (16)	0.0036 (17)
C6	0.0431 (17)	0.0526 (19)	0.0360 (17)	−0.0029 (14)	−0.0102 (13)	0.0059 (13)
C7	0.059 (2)	0.060 (2)	0.082 (3)	0.0044 (17)	0.013 (2)	−0.024 (2)
C8	0.0496 (18)	0.0484 (19)	0.055 (2)	0.0031 (14)	−0.0012 (16)	−0.0073 (15)
C9	0.0500 (18)	0.0488 (19)	0.0435 (18)	0.0055 (14)	0.0069 (15)	−0.0042 (14)
C10	0.0403 (15)	0.0357 (15)	0.0356 (15)	0.0025 (12)	0.0003 (12)	0.0038 (12)
Cl1	0.0624 (6)	0.0570 (6)	0.0882 (7)	0.0174 (4)	−0.0131 (5)	−0.0005 (5)
Cl2	0.0583 (5)	0.0617 (5)	0.0438 (4)	0.0026 (4)	0.0076 (4)	0.0148 (4)
Cl3	0.0789 (6)	0.0675 (6)	0.0597 (6)	−0.0330 (5)	−0.0095 (5)	0.0051 (5)
Cl4	0.0683 (6)	0.0638 (6)	0.0397 (4)	−0.0043 (4)	0.0026 (4)	−0.0073 (4)
Fe1	0.0475 (3)	0.0410 (3)	0.0368 (3)	−0.00288 (18)	−0.00255 (19)	0.00161 (18)
N1	0.0337 (12)	0.0370 (13)	0.0294 (12)	0.0002 (10)	0.0008 (10)	0.0057 (9)

### Geometric parameters (Å, º)

**Table:**

C1—C2	1.488 (4)	C6—H6B	0.9700
C1—H1A	0.9600	C7—C8	1.498 (5)
C1—H1B	0.9600	C7—H7A	0.9600
C1—H1C	0.9600	C7—H7B	0.9600
C2—N1	1.498 (3)	C7—H7C	0.9600
C2—H2A	0.9700	C8—C9	1.501 (4)
C2—H2B	0.9700	C8—H8A	0.9700
C3—C4	1.497 (4)	C8—H8B	0.9700
C3—H3A	0.9600	C9—C10	1.492 (4)
C3—H3B	0.9600	C9—H9A	0.9700
C3—H3C	0.9600	C9—H9B	0.9700
C4—N1	1.504 (3)	C10—N1	1.496 (3)
C4—H4A	0.9700	C10—H10A	0.9700
C4—H4B	0.9700	C10—H10B	0.9700
C5—C6	1.486 (4)	Cl1—Cl1	0.0000 (18)
C5—H5A	0.9600	Cl1—Fe1	2.1804 (10)
C5—H5B	0.9600	Cl2—Fe1	2.1611 (9)
C5—H5C	0.9600	Cl3—Fe1	2.1695 (10)
C6—N1	1.504 (3)	Cl4—Fe1	2.1823 (10)
C6—H6A	0.9700	Fe1—Cl1	2.1804 (10)
			
C2—C1—H1A	109.5	C8—C7—H7C	109.5
C2—C1—H1B	109.5	H7A—C7—H7C	109.5
H1A—C1—H1B	109.5	H7B—C7—H7C	109.5
C2—C1—H1C	109.5	C7—C8—C9	112.6 (3)
H1A—C1—H1C	109.5	C7—C8—H8A	109.1
H1B—C1—H1C	109.5	C9—C8—H8A	109.1
C1—C2—N1	116.3 (2)	C7—C8—H8B	109.1
C1—C2—H2A	108.2	C9—C8—H8B	109.1
N1—C2—H2A	108.2	H8A—C8—H8B	107.8
C1—C2—H2B	108.2	C10—C9—C8	110.4 (3)
N1—C2—H2B	108.2	C10—C9—H9A	109.6
H2A—C2—H2B	107.4	C8—C9—H9A	109.6
C4—C3—H3A	109.5	C10—C9—H9B	109.6
C4—C3—H3B	109.5	C8—C9—H9B	109.6
H3A—C3—H3B	109.5	H9A—C9—H9B	108.1
C4—C3—H3C	109.5	C9—C10—N1	116.6 (2)
H3A—C3—H3C	109.5	C9—C10—H10A	108.1
H3B—C3—H3C	109.5	N1—C10—H10A	108.1
C3—C4—N1	115.4 (3)	C9—C10—H10B	108.1
C3—C4—H4A	108.4	N1—C10—H10B	108.1
N1—C4—H4A	108.4	H10A—C10—H10B	107.3
C3—C4—H4B	108.4	Cl1—Cl1—Fe1	0 (10)
N1—C4—H4B	108.4	Cl2—Fe1—Cl3	109.72 (4)
H4A—C4—H4B	107.5	Cl2—Fe1—Cl1	109.41 (5)
C6—C5—H5A	109.5	Cl3—Fe1—Cl1	109.55 (5)
C6—C5—H5B	109.5	Cl2—Fe1—Cl1	109.41 (5)
H5A—C5—H5B	109.5	Cl3—Fe1—Cl1	109.55 (5)
C6—C5—H5C	109.5	Cl1—Fe1—Cl1	0.00 (7)
H5A—C5—H5C	109.5	Cl2—Fe1—Cl4	108.71 (4)
H5B—C5—H5C	109.5	Cl3—Fe1—Cl4	110.18 (5)
C5—C6—N1	115.2 (2)	Cl1—Fe1—Cl4	109.26 (5)
C5—C6—H6A	108.5	Cl1—Fe1—Cl4	109.26 (5)
N1—C6—H6A	108.5	C10—N1—C2	111.0 (2)
C5—C6—H6B	108.5	C10—N1—C6	111.5 (2)
N1—C6—H6B	108.5	C2—N1—C6	106.5 (2)
H6A—C6—H6B	107.5	C10—N1—C4	106.3 (2)
C8—C7—H7A	109.5	C2—N1—C4	110.8 (2)
C8—C7—H7B	109.5	C6—N1—C4	110.8 (2)
H7A—C7—H7B	109.5		

### Hydrogen-bond geometry (Å, º)

**Table:**

*D*—H···*A*	*D*—H	H···*A*	*D*···*A*	*D*—H···*A*
C3—H3*B*···Cl3^i^	0.96	2.87	3.790 (4)	162
C4—H4*A*···Cl1	0.97	2.92	3.859 (3)	163

Symmetry code: (i) *x*+1, *y*, *z*.
